# Holistic Skincare Approach of Cleanse–Treat–Moisturize–Protect (CTMP®) in Atopic Dermatitis Management: Indian Perspectives, Evidence, and Future Directions

**DOI:** 10.7759/cureus.105971

**Published:** 2026-03-27

**Authors:** Sanjay Choudhary, Dinkar Sadana, Vijayabhaskar C, Dyotona Sen, Soma Iyer

**Affiliations:** 1 Medicine, Galderma India Pvt Ltd., Mumbai, IND; 2 Dermatology, Dr. Sadana’s Skin clinic and Laser centre, Patiala, IND; 3 Dermatology, Stanley Medical College, Chennai, IND

**Keywords:** atopic dermatitis, ceramide-based moisturization, cleanse-treat-moisturize-protect protocol, ctmp, dermatologist, epidermal barrier dysfunction, holistic skincare

## Abstract

Atopic dermatitis (AD) is a chronic, relapsing inflammatory dermatosis characterized by epidermal barrier dysfunction, immune dysregulation, and significant impairment in quality of life, particularly among pediatric populations. Although topical anti-inflammatory therapies remain central to acute flare control, growing evidence supports barrier-directed skincare as a fundamental component of long-term disease management. This narrative review synthesizes current evidence on cleansing, moisturization, photoprotection, and preventive strategies in AD, with specific consideration of pediatric care and real-world challenges within the Indian healthcare context. Clinical studies consistently demonstrate that regular emollient use enhances skin hydration, accelerates barrier recovery, alleviates symptom burden, and improves quality of life, with additive benefits when combined with topical corticosteroids. National guidance further emphasizes age-appropriate bathing practices, including delayed bathing in low-birth-weight infants and gentle cleansing followed by prompt emollient application. Despite these recommendations, Indian surveys reveal persistent gaps in caregiver awareness, delayed diagnosis, intermittent moisturizer use, and suboptimal adherence driven by cost constraints, loss to follow-up, and preference for alternative therapies. While emerging modalities, including biomimetic formulations, digital health platforms, and personalized skincare, offer potential future directions, current evidence remains largely exploratory, with limited data on cost-effectiveness and real-world implementation. Overall, barrier-focused skincare represents an important adjunct to pharmacologic therapy in AD; however, effective integration into Indian clinical practice will require strengthened caregiver education, context-specific guidance, and system-level strategies addressing accessibility and adherence, supported by future pragmatic trials and epidemiological studies to enable scalable, evidence-based care.

## Introduction and background

Introduction

Atopic dermatitis (AD) is a chronic, relapsing inflammatory skin disorder that substantially impairs the quality of life and places a considerable burden on healthcare systems [1]. Typically present in infancy or childhood, AD is characterized by pruritus, xerosis, and recurrent flares with erythema, oozing, itching, and sleep disturbances, which add to the disease burden [2]. Globally, AD affects 20% of children and 5% of adults worldwide [3], causing social isolation and psychological stress [4]. Approximately 80% of cases develop in early childhood, with nearly 60% achieving remission by adolescence [5]. The reported prevalence of AD in India between 2011 and 2021 ranged from 0.98% to 9.2% across pediatric and adult populations [6]. The Indian consensus describes AD as a common, chronic inflammatory skin disorder of childhood that has a substantial impact on the physical, psychological, and social well-being of the affected children and their families [7].

AD is not limited to cutaneous disease and is accompanied by systemic and psychosocial consequences. Persistent pruritus, sleep disturbance, and recurrent flares impair academic or work performance and impose caregiver strain in pediatric cases [8,9]. AD arises from interconnected epidermal barrier dysfunction, immune dysregulation, gut microbial imbalance, and neuroimmune signaling that together drive disease onset, chronicity, and relapse. Central barrier defects, such as filaggrin deficiency, ceramide depletion, and elevated skin pH, lead to increased transepidermal water loss (TEWL), impaired antimicrobial defense, and enhanced allergen and microbial penetration. Barrier disruption triggers epithelial alarmins and type 2-skewed immune responses (interleukin (IL)-4, IL-13, IL-31), which further damage barrier integrity, sustain inflammation, and induce pruritus, with additional T-helper (Th)22/Th1/Th17 pathways emerging in chronic disease. Cutaneous dysbiosis and neuroimmune itch-scratch interactions amplify inflammation and barrier breakdown, explaining the relapsing course, infection susceptibility, and clinical heterogeneity of AD [10-12].

The pharmacological management of AD relies mainly on topical corticosteroids and calcineurin inhibitors [13]. However, evolving paradigms emphasize long-term non-pharmacological strategies, particularly structured skincare. The skincare approach of CTMP^®^ or Cleanse, Treat, Moisturize, and Protect supports AD care, with moisturization regarded as the primary non-pharmacological intervention for barrier repair and flare prevention [14].

In India, the uptake of holistic skincare frameworks such as CTMP^®^ remains limited due to challenges like widespread hard‑water exposure, deeply rooted cultural habits, economic constraints, and low patient awareness, especially across rural and semi‑urban populations [12].

A key unmet need in AD management is the lack of consistent long-term strategies that address both skin barrier dysfunction and patient adherence, despite the availability of pharmacological therapies. There is limited integration of structured adjunctive care, such as the CTMP^®^ approach, including the use of filaggrin-based moisturizing technologies that directly target barrier impairment. Moreover, awareness and implementation of CTMP^®^ across India remain insufficient, highlighting the need for strengthened clinician guidance, policy implementation, and patient education to support holistic, sustained disease management.

## Review

Methodology

This narrative review was compiled through searches conducted in PubMed/MEDLINE, EMBASE, Scopus, Web of Science, and the Cochrane Library from January 2004 through December 2026, followed by manual screening of reference lists and Indian dermatology guidelines to ensure region-specific relevance. The search strategy combined controlled vocabulary and free‑text terms related to AD, skin barrier impairment, cleansing practices, syndet formulations, moisturizers (ceramides, humectants, occlusives), photoprotection (sunscreens, ultraviolet exposure), and pharmacologic therapies (topical corticosteroids, calcineurin inhibitors, biologics, Janus kinase (JAK) inhibitors), as well as Indian or Asia‑Pacific care practices. We included peer-reviewed clinical studies, reviews, guidelines, consensus statements, and mechanistic research, and excluded non-peer-reviewed commentary and promotional content. Given heterogeneity in study designs and outcome measures (e.g., hydration, trans epidermal water loss, Eczema Area and Severity Index (EASI) scores, pruritus, quality of life), findings were synthesized descriptively rather than pooled statistically. All quantitative data were verified against primary publications.

Pathophysiology and skin barrier dysfunction in AD

Core barrier abnormalities include filaggrin deficiency, ceramide depletion, and elevated skin surface pH. Loss-of-function mutations in the filaggrin gene reduce natural moisturizing factors such as pyrrolidone carboxylic acid and urocanic acid, resulting in increased TEWL and xerosis [12]. Reduced stratum corneum ceramides disrupt lamellar bilayer organization, diminish water retention, and compromise barrier integrity [10]. Elevated skin surface pH further impairs lipid-processing enzymes and antimicrobial peptide activity, weakening epidermal defenses and facilitating microbial colonization [15].

Barrier disruption activates keratinocytes to release epithelial-derived alarmins, including thymic stromal lymphopoietin (TSLP), IL-25, and IL-33, initiating downstream immune cascades. These mediators promote activation of dendritic cells, innate lymphoid cells, and T lymphocytes, driving dominant type 2 immune polarization, characterized by IL-4, IL-13, and IL-31 signaling. IL-4 and IL-13 impair keratinocyte differentiation and lipid synthesis, perpetuating barrier dysfunction, while IL-31 mediates pruritus through sensory neuron activation. With chronic disease, additional immune pathways emerge, including Th22-mediated epidermal hyperplasia via IL-22 and variable Th1 and Th17 contributions, contributing to clinical heterogeneity and providing the mechanistic basis for newer targeted therapies [10].

Cutaneous dysbiosis both reflects and amplifies barrier-immune perturbations. Staphylococcus aureus colonization occurs in 70-90% of AD lesions and exacerbates inflammation through superantigen activity while delaying barrier repair [15]. Beyond single-pathogen dominance, AD is characterized by increased S. aureus, Staphylococcus spp., and Corynebacterium, with reduced Streptococcus, Propionibacterium (Cutibacterium), Acinetobacter, and Staphylococcus epidermidis. Restoration of commensal species, including S. epidermidis, Streptococcus, Corynebacterium, and Propionibacterium, is associated with clinical improvement, collectively modulating inflammatory tone and epidermal barrier stability [10].

Neuroimmune interactions further perpetuate disease activity. Cytokines and pruritogenic mediators released by immune cells and keratinocytes activate peripheral sensory neurons, initiating an itch-scratch cycle that disrupts the epidermal barrier and intensifies inflammation, while reciprocal neural signaling modulates immune responses [11,16]. Collectively, this integrated barrier-immune-microbiome-neural axis explains the hallmark clinical features of AD, including xerosis, recurrent flares, pruritus, susceptibility to infection, and heterogeneous therapeutic responses, underscoring the importance of management strategies that address barrier repair, immune modulation, and microbial balance alongside pharmacologic intervention.

Guideline-based management of AD

Current expert recommendations advocate for a personalized, stepwise management plan for AD that integrates both pharmacologic and supportive non‑pharmacologic measures. The overall aim is to reduce inflammation while rebuilding and maintaining the integrity of the skin barrier. Core components of evidence‑driven care include appropriate cleansing practices, targeted anti‑inflammatory interventions, regular moisturization, and protective strategies collectively represented by the CTMP^®^ approach [14].

Cleansing in AD

Role of cleansers in barrier protection: Cleansing plays a critical role in AD care by helping eliminate sweat, debris, microbes, allergens, and remnants of topical products [17]. However, improper habits, such as frequent hot-water bathing, using alkaline or harsh soaps, or aggressive scrubbing, can further impair the already vulnerable epidermal barrier, heightening TEWL and worsening xerosis and itch [18-20]. Because the AD skin barrier is intrinsically compromised, with elevated surface pH, reduced ceramides, and disrupted lipid lamellae, it is particularly prone to surfactant-related injury [9,14]. Traditional soaps and certain cleansers, which often contain strong surfactants and lack physiological pH, can deplete intercellular lipids, raise skin pH, and increase TEWL, effects most pronounced with anionic and cationic surfactants. In contrast, syndet formulations using mild non‑ionic or amphoteric surfactants (e.g., alkyl polyglucosides, cocamidopropyl betaine) at skin‑compatible pH (≈5.0-5.5) better maintain stratum corneum structure, lipid integrity, microbiome balance, and cleansing efficacy [21].

Ideal cleansing agents and their features and evidence: Traditional alkaline soaps (pH ~10.2) can disrupt the stratum corneum, deplete intercellular lipids, and further compromise the skin barrier in AD [22]. Accordingly, clinical guidance recommends non‑soap surfactants and syndet cleansers with neutral to mildly acidic pH, which demonstrate superior tolerability compared with bar soaps. In one study, 41% of soap users discontinued due to erythema, whereas no discontinuations occurred with a non‑soap cleansing lotion [23-25].

Gentle, soap‑free cleansing also enhances stratum corneum hydration and improves the effectiveness of topical anti‑inflammatory therapies, with syndets accelerating clinical improvement and supporting overall barrier repair [7]. Evidence from randomized controlled trials consistently favours syndet-based formulations because they help maintain near‑physiological pH, minimise lipid and protein loss, and frequently incorporate humectants or barrier-supportive lipids [23,26-29]. Pediatric and Asia-Pacific consensus further recommend cleansers that are soap-free, fragrance-free, hypoallergenic, mildly acidic, effectively and gently cleanse dry, itchy skin, provide immediate and long-lasting hydration, and always followed by moisturization, highlighting cleanser choice as an important decision integral to the CTMP^®^ regimen in AD management [14,30]. In atopic skin, gentle cleansers containing natural moisturizing factors (e.g., filaggrin breakdown products) were well tolerated, reduced itch, and improved quality of life [17]. In-use studies of high-emollient cleanser formulations in dry, atopic-prone skin have demonstrated good dermatologic tolerability with improvements in objective severity scores (SCORing Atopic Dermatitis or SCORAD) and skin hydration. Controlled trials of syndet body wash regimens in children with AD in remission have evaluated clinical signs and symptoms over 12 weeks, justifying the incorporation of skin-restoring cleansers as part of evidence-based AD management while enabling barrier protection and symptomatic relief [31-33]. Emerging data further highlight the important role of soap‑free cleansers enriched with humectants, filaggrin‑derived components, or lipid‑deposition systems as integral elements of the CTMP^®^ regimen, improving xerosis, pruritus, and patient‑reported outcomes [14,17,30,34]. In-use studies of high-emollient cleanser formulations in dry, atopic-prone skin have demonstrated good dermatologic tolerability with improvements in objective severity scores (SCORAD) and skin hydration [32].

Bathing once daily or on alternate days for five to 10 minutes using lukewarm water is recommended, since longer durations or hot water can worsen barrier impairment. Applying an emollient within three to five minutes after bathing, known as the “soak and seal” method, helps maximize hydration and supports barrier repair. In resource‑constrained regions such as India, substituting traditional alkaline soaps with affordable syndet cleansers and educating caregivers to avoid prolonged hot baths are important for improving adherence and treatment outcomes [14,26,27,29,34-36].

Indian cleansing practices are further influenced by hard water, which interacts with soap surfactants to increase skin pH and irritancy [37], along with cultural habits such as frequent bathing, vigorous scrubbing, herbal powders, and pre‑bath oil application. Although oil massage offers partial occlusion, it may raise infection risk if the oil is contaminated or not adequately rinsed. Patient counselling should reinforce short lukewarm baths, gentle use of mild cleansers, avoidance of harsh soaps, and immediate moisturization after bathing [38]. Ensuring access to affordable and culturally acceptable skincare products remains essential for long‑term adherence and disease control in Indian settings [39].

Evidence specific to Indian populations regarding sustained reductions in TEWL and long‑term benefits remains limited, and real‑world adherence is often affected by socioeconomic constraints and product accessibility. These gaps highlight the need for regional quantitative studies focused on barrier outcomes and culturally aligned care rather than relying solely on generalized recommendations [40,41].

Treatment of AD

While cleansing and moisturization primarily focus on restoring the barrier and maintaining hydration, pharmacological agents are critical for modulating the underlying immune dysregulation during acute flares or chronic severe disease.

Role of topical corticosteroids (TCS): TCS remain the first‑line anti‑inflammatory treatment for acute AD flares because they effectively suppress cytokine‑driven inflammation, alleviate erythema and pruritus, and restore overall skin comfort [42]. Their use is guided by potency, patient age, anatomical site, and disease severity, with preparations categorized into low, moderate, high, and ultra‑high potency groups. Low-potency TCS, such as hydrocortisone, are preferred for pediatric patients with AD and plaque psoriasis, where wider safety margins are essential, while moderate- to high-potency agents are reserved for steroid-responsive dermatoses [43]. Desonide 0.05% has demonstrated efficacy in reducing erythema, pruritus, and lesion severity in mild-to-moderate AD, with low systemic absorption and minimal risk of cutaneous atrophy when used appropriately [44], supporting its use in children and delicate skin sites. Despite their efficacy, concerns about potential adverse effects, including skin atrophy, striae, and hypothalamic-pituitary-adrenal axis suppression, often contribute to corticosteroid phobia and reduce adherence [45]. Careful selection of potency, controlled duration of use, and concurrent barrier‑supportive skincare are central to mitigating these risks.

Clinical evidence indicates that low‑potency corticosteroids, such as hydrocortisone and desonide, have favourable safety profiles when applied intermittently [44]. However, variability in study designs, treatment durations, and patient populations limits direct comparison across individual molecules. Overall, effective AD management focuses not on selecting a specific corticosteroid molecule but on ensuring appropriate potency choice, safe and guided usage, and consistent moisturization to enhance therapeutic response and reduce cumulative steroid exposure.

Optimizing steroid use through CTMP^®^ integration: Combining TCS with regular moisturization has been shown to improve treatment outcomes. In a randomized study, adjunctive use of a ceramide and filaggrin-derived component-enriched moisturizer with corticosteroids improved skin hydration and accelerated EASI score improvement compared with corticosteroid monotherapy [18]. This combined regimen also reduces steroid reliance and supports better adherence, underscoring that moisturization is an essential component of pharmacologic management rather than an optional adjunct [34].

Topical calcineurin inhibitors (TCIs): TCIs, such as tacrolimus and pimecrolimus, act by inhibiting calcineurin-dependent T cell activation and reducing cytokine release. TCIs can be useful for sensitive areas (face, eyelids, flexures) where prolonged high-potency corticosteroid use risks atrophy and for patients who are steroid-averse or have contraindications to corticosteroids [44].

Gentle cleansing before TCI application optimizes absorption, while concurrent moisturization reduces the burning or stinging sensations often reported during the initial treatment, thereby improving tolerability [46]. Tacrolimus is generally more effective in moderate to severe disease, while pimecrolimus is suited to milder forms. Long‑term safety has been debated since the FDA black box warning in 2006, though subsequent studies have not confirmed a causal malignancy risk; appropriate use is considered safe, with caution in children and prolonged widespread application. Evidence linking skincare practices directly to TCI pharmacodynamics is limited, so integration within CTMP^®^ should be seen as supportive rather than prescriptive. In stepwise therapy, TCIs are positioned as second-line agents after corticosteroids [47-50]. Integrating TCIs within the holistic skincare (CTMP^®^) principle ensures barrier recovery along with inflammation control.

Therapeutic Role of Moisturizers in AD Management

Clinical implications for skincare intervention: Barrier impairment and immune activation in AD are closely interconnected, creating the need for both anti‑inflammatory therapy and consistent barrier‑supportive skincare [35]. Regular moisturization plays a central role in AD management because emollients enhance hydration, replenish epidermal lipids, help normalize skin surface pH, and reduce penetration of irritants and allergens [14]. A range of clinical studies evaluating formulations such as ceramide‑dominant, petrolatum‑based, glycerin‑rich, and urea‑containing moisturizers have demonstrated improvements in skin hydration, reductions in TEWL, and enhanced patient comfort when used consistently [51].

Adjunctive moisturization with TCS may improve therapeutic response and decrease overall steroid requirements, although outcomes vary depending on the product type, disease severity, patient age, and adherence [52]. Early preventive studies have also suggested that emollient use in high‑risk neonates may lower the likelihood of developing AD [53].

Collectively, these findings underscore the importance of maintaining barrier‑supportive skincare throughout all phases of AD. Presenting routines in a simple structure such as CTMP^®^, can help patients and caregivers understand the intended sequence of therapy application, although this framework serves primarily as a practical guide rather than a formally validated protocol.

Moisturization in long-term management of AD: Moisturization is a central component of AD care and represents the leading non‑pharmacological approach for restoring barrier integrity, maintaining hydration, and preventing flares [54]. While anti‑inflammatory therapies such as corticosteroids and biologics target immune dysregulation, moisturizers address the underlying structural abnormalities of the epidermis, which are fundamental to AD pathogenesis [55]. Barrier impairment in AD results in elevated TEWL, reduced natural moisturizing factors (NMF), and disrupted lipid organization, changes that promote increased allergen entry, susceptibility to microbial colonization, especially Staphylococcus aureus, and amplification of cutaneous inflammation [56]. Consistent use of moisturizers helps break this cycle by improving barrier repair and enhancing clinical outcomes when used alongside pharmacologic treatments [14,28].

Types of moisturizers: Moisturizers support hydration and barrier repair through three key mechanisms: occlusion, emollience, and humectancy. Occlusive agents create a hydrophobic layer that limit water loss; petrolatum, dimethicone, and mineral oil are commonly used for this purpose and are particularly effective in marked xerosis, although some patients may find them less cosmetically acceptable in hot or humid climates. Emollients, including fatty acids, cholesterol, and triglycerides, soften the skin by filling intercellular spaces, whereas humectants such as glycerin, urea, and hyaluronic acid attract and retain water within the stratum corneum to enhance hydration and elasticity [16].

As mentioned in the consensus and expert opinion, an ideal/recommended moisturizer for AD should protect and restore skin barrier function, improve stratum corneum hydration, and provide sustained hydration after a single application. It should soothe, moisturize, and provide immediate relief from itching, redness, and irritation, improve local tolerance to topical treatment without affecting its efficacy, and be hypoallergenic, non-acnegenic, fragrance free, with potential anti-inflammatory and antioxidant properties [14].

Contemporary barrier‑repair formulations typically combine these mechanisms and include additional activities aimed at reinforcing barrier function. Ceramides and their precursors help restore the lamellar lipid structure of the stratum corneum, while filaggrin‑derived NMF components such as pyrrolidone carboxylic acid and arginine augment hydration and reduce TEWL. Panthenol contributes to hydration and supports epidermal repair, and niacinamide promotes ceramide synthesis while providing anti‑inflammatory benefits. Glycerin serves as a well‑established humectant, and peptide complexes such as Adresyl (AD-RESYL®, SILAB Softcare, France) have been reported to improve epidermal differentiation and long‑term barrier integrity [28,57]. Additionally, Adresyl, helps in maintaining microbiome balance by enhancing microbial resilience, inhibiting pathogenic overgrowth, supporting short-chain fatty acid production, and strengthening intestinal barrier and immune function [58]. Ceramide- and NMF-containing moisturizers address barrier defects in AD by replenishing deficient lipids and improving stratum corneum integrity, with meta-analyses showing greater improvements in SCORAD versus conventional emollients. In a randomized controlled trial in children with AD, ceramide-based and paraffin-based moisturizers both significantly improved disease severity and TEWL, with comparable SCORAD and quality-of-life outcomes. Ceramide and filaggrin-derived component-enriched moisturizer formulation demonstrated significant reductions in barrier dysfunction, pruritus, and symptom scores with good tolerability in AD-prone skin. Overall, ceramide and filaggrin-derived component-enriched moisturizers are effective adjuncts in AD due to proven barrier repair, symptom reduction, and improved patient-reported outcomes [59-62].

In evaluator-blinded, randomized, and intra-individual comparison trials, ceramide and filaggrin-derived component-enriched moisturizer formulation (Cetaphil Restoraderm™ Skin Restoring Body Moisturizer; Galderma, Zug, Switzerland) demonstrated superior performance compared with other moisturizers in improving skin hydration and supporting barrier recovery in individuals with AD. A single application produced a rapid and sustained increase in skin hydration for up to 24 hours, with significantly greater hydration levels than comparator moisturizers at multiple time points. In a barrier disruption model, repeated application of this moisturizer led to faster restoration of skin barrier function and consistently higher hydration compared with reference products. These findings suggest that ceramide and filaggrin-derived component-enriched moisturizer provides enhanced moisturization and barrier-support benefits relative to other emollient formulations [18].

Evidence for specific moisturizing ingredients varies, with clinical benefits influenced by formulation characteristics, concentration, and adherence. Comparative studies have noted differences between ceramide‑dominant lipid creams, urea‑based moisturizers that provide both humectant and keratolytic effects, and petrolatum‑rich ointments that offer strong occlusion but may have lower cosmetic acceptability.

Phototherapy and Photoprotection in AD

In AD, exposure to ultraviolet rays (UV) has a dual role. Controlled therapeutic phototherapy can be beneficial in chronic or refractory cases; however, unprotected excessive UV exposure may impair skin barrier function, increase oxidative stress, and trigger disease flares. It may also contribute to pigmentary alterations, particularly post-inflammatory hyperpigmentation in individuals with Indian skin types [63-65]. Due to India’s consistently high UV index, regular photoprotection is advised even during maintenance phases [30]. For sensitive skin, broad‑spectrum and fragrance‑free sunscreens are recommended, with physical filters such as zinc oxide and titanium dioxide offering favorable tolerability, and hybrid formulations improving cosmetic acceptance without reducing protection [66,67]. Recommended application practices include applying moisturizer first, followed by sunscreen after 15 to 20 minutes, and reapplying every two to three hours during outdoor exposure [17]. While narrowband UVB phototherapy should only be used under clinical supervision because of cumulative UV risks [65], daily photoprotection remains essential to reduce UV‑induced oxidative stress and pigmentary alterations that worsen barrier dysfunction and quality of life [30]. Educational gaps persist in India due to cost concerns and misconceptions about photoprotection in darker skin, and year‑round strategies such as protective clothing, wide‑brim hats, and sunglasses are encouraged, especially for children who are more susceptible to UV‑related barrier injury [68-70]. Within the CTMP^®^ sequence (cleanse → treat → moisturize → protect), sunscreens containing hydrating and barrier‑supportive ingredients such as glycerin, and panthenol may improve adherence, and combination products like moisturizers with sun protection factor (SPF) provide practical options for patients who prefer simplified routines [17,71].

In the Indian population, where Fitzpatrick phototypes IV to VI are common, accurate phototyping remains essential because, despite greater melanin‑mediated protection, these skin types are still susceptible to UV‑induced tanning, pigmentary disorders, and a heightened risk of dyspigmentation during light‑based procedures [72].

Phototherapy is effective in managing AD, either alone or with emollients and topical corticosteroids. Narrowband ultraviolet B (NB‑UVB; 311-313 nm) is widely used as a first‑line option for pediatric dermatoses due to its efficacy and safety. However, data on Indian skin types remain limited. Experts recommend NB‑UVB for selected cases such as chronic eczema or palmo‑plantar involvement, but caution against its use during acute flares because of risks of worsening erythema, burning, and pruritus [73].

Flare Prevention

Evidence for barrier repair and flare prevention: One evaluator-blinded, randomized intra-individual study demonstrated that ceramide and filaggrin-derived component-enriched moisturizer (Cetaphil Restoraderm™ Skin Restoring Body Moisturizer) produces sustained skin hydration for up to 24 hours and accelerates recovery with significant improvements in pruritus, quality of life, and patient satisfaction after four weeks of twice-daily application. When used adjunctively with topical corticosteroids, moisturization was associated with faster reductions in disease severity at days seven, 14, and 21 and greater hydration than steroid monotherapy (P<0.05), supporting barrier-directed skincare as a clinically relevant adjunct for flare control. The formulations evaluated incorporated ceramides, filaggrin breakdown products, and a combination of humectants, emollients, and occlusives to support the epidermal barrier function and hydration [18].

Early identification of persistent itch, xerosis, and recurrent eczematous lesions enables timely intervention and may improve outcomes [74]. Preventive strategies emphasize emollient use from infancy, avoidance of harsh soaps and irritants, and early management of allergic sensitizations to reduce flare risk [75-78]. Caregiver education improves recognition of warning signs and adherence to preventive measures, with demonstrated benefits from structured tools and counseling [79-81].

During acute flares, a severity‑stratified step‑up approach is recommended, using emollients with low‑ to mid‑potency TCS for mild flares and short courses of higher‑potency corticosteroids for moderate to severe flares, and transition to proactive maintenance within a CTMP^®^ framework, supported by caregiver guidance [82]. Antimicrobials are reserved for clinically evident secondary infection, prolonged use is discouraged due to resistance and microbiome disruption, and antiseptic options such as diluted bleach baths may benefit selected patients. Wet‑wrap therapy can rapidly reduce severity in moderate to severe flares but is limited by caregiver burden, climate‑related discomfort, and adherence challenges, and uptake of TCIs and proactive regimens may be constrained by cost, access, and safety considerations requiring individualized counseling [83].

Photoprotection functions as a chronic supportive measure rather than an acute flare intervention, especially in skin of color where UV and visible light mainly contribute to pigmentary sequelae. Protective clothing and broad‑spectrum sunscreens (SPF ≥30, UVA‑PF/SPF ≥2/3, tinted formulations) are recommended to prevent UV‑induced oxidative stress and pigmentation that worsen barrier function and quality of life [73,82,84].

Maintenance and flare prevention: Daily moisturization remains fundamental in preventing flares in AD, and structured care plans such as CTMP^®^ may help support routine skincare implementation in selected patients, although their impact on adherence and long‑term control varies across different populations and clinical environments [85]. Within established stepwise management frameworks, proactive use of TCS or TCIs following flare resolution is recommended for individuals with recurrent episodes or site‑specific relapses. When combined with consistent emollient therapy, this proactive strategy has been shown to lower relapse rates and reduce overall disease burden [20,73,82].

Seasonal and lifestyle modification

CTMP^®^ routines should be adjusted according to seasonal shifts and cultural habits. During the monsoon and winter months, when xerosis is more pronounced, patients benefit from thicker emollients and occlusive barrier creams to maintain adequate hydration [28,57]. In contrast, summer conditions call for lighter, non‑greasy formulations alongside consistent photoprotection to address heat‑ and UV‑related triggers [86]. Awareness of cultural practices common in India, such as vigorous scrubbing, is also important, as these behaviors can disrupt the skin barrier and should be discouraged in patients with AD [87].

Challenges

Caregiver Education and Behavioral Reinforcement

Caregivers of children with AD experience a substantial multidimensional burden that extends beyond day‑to‑day treatment tasks. Greater disease severity correlates with disrupted sleep, psychological stress, impaired functioning, and financial pressures, all of which undermine adherence and long‑term control [88]. Non‑adherence is frequently driven by regimen complexity, forgetfulness, cost, and fear of adverse effects, particularly steroid phobia. Misinterpretation of instructions, uncertainty about topical potencies, and suboptimal clinician-caregiver communication further reduces correct use, with objective assessments often revealing underapplication despite high self‑reported compliance. These factors collectively diminish treatment effectiveness and sustained control even when advanced therapies are available [89].

Digital adherence tools, including mobile apps and reminder systems, can improve compliance in pediatric AD, particularly when used by caregivers [90]. However, real‑world implementation is limited by low digital literacy, caregiver time constraints, infrastructure and usability barriers, suboptimal integration with existing platforms, and privacy concerns-factors often underrepresented in efficacy‑focused trials. Accordingly, these tools should be considered context dependent and adjunctive rather than standalone solutions [91].

CTMP^®^ Integration in the Indian Healthcare Ecosystem

Infant skin care in India reflects a convergence of traditional customs and modern practices, many of which lack robust scientific validation. This heterogeneity, coupled with the absence of unified national guidelines, highlights the need for culturally sensitive yet evidence-based recommendations to ensure safety and optimize outcomes [92]. National child health platforms such as Integrated Management of Neonatal and Childhood Illnesses (IMNCI) and Rashtriya Bal Swasthya Karyakram prioritize early identification of pediatric conditions but offer limited operational guidance on barrier-focused skincare, underscoring a gap between policy and clinical practice [93,94]. Within routine newborn care, light oil massage followed by warm water sponging is commonly recommended, with strict avoidance of oil instillation into the nose or ears.

Real-world adherence challenges further complicated AD management. A 2025 survey of Indian dermatologists reports that non-adherence to AD therapy is primarily driven by cost of treatment, loss to follow-up, and preference for alternative medicine [95]. Complementary parent survey data demonstrate limited awareness and persistent misconceptions regarding AD, including under-recognition of moisturizers as essential therapy despite general acknowledgment of their importance [23]. Although Indian expert consensus statements identify moisturizers as a cornerstone of AD management, clinical practice surveys indicate that they are frequently used intermittently or restricted to flare periods [7]. Current Indian AD guidelines and expert recommendations advocate daily bathing with lukewarm water followed by prompt, generous emollient application (e.g., “soak and smear”) to support barrier repair and reduce dependence [73]. Additionally, a 2025 national dermatologist survey highlights delayed diagnosis and inappropriate treatment as key systemic gaps, emphasizing the need for enhanced training and awareness at primary care and community levels to improve real-world AD outcomes [95].

Future directions

Future advances in AD management in India should prioritize implementation-focused research that addresses persistent challenges such as delayed diagnosis, inconsistent follow-up, cost constraints, and caregiver knowledge gaps. Emerging approaches, including biomimetic barrier-repair formulations and personalized skincare guided by genetic or microbiome profiling, are supported largely by early-stage evidence, underscoring the need for larger, population-based clinical trials before these strategies can be adopted in routine care. Moreover, given the established role of filaggrin deficiency in AD pathophysiology and its use as a diagnostic biomarker, filaggrin-based or filaggrin-enhancing moisturizers demonstrate promising efficacy in improving barrier function and should be considered for wider clinical application [96].

Digital health interventions, such as tele dermatology and adherence-support applications, demonstrate feasibility but continue to face limitations related to digital inequity, infrastructural variability, and evolving regulatory frameworks [97,98]. Artificial-intelligence-driven personalization similarly remains exploratory and requires rigorous clinical validation before real-world deployment [99]. Key priorities moving forward include generating robust Indian epidemiological datasets; conducting cost-effectiveness analyses for long-term moisturization and anti-inflammatory treatment strategies; and performing implementation studies that integrate guideline-based skincare into primary care and national child health programs [73,100]. Strengthening caregiver education tailored to literacy levels is essential, and Indian AD expert consensus documents consistently emphasize that treatment regimens must be adapted to climatic conditions, age, disease severity, and socioeconomic context-highlighting the need for region-specific care pathways rather than uniform, one-size-fits-all protocols [101].

Illustrative clinical vignette

**Figure 1:**
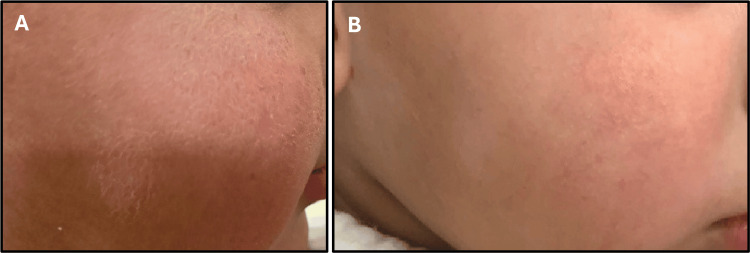
Facial atopic dermatitis before (A) and after treatment (B) showing reduction in erythema

**Figure 2:**
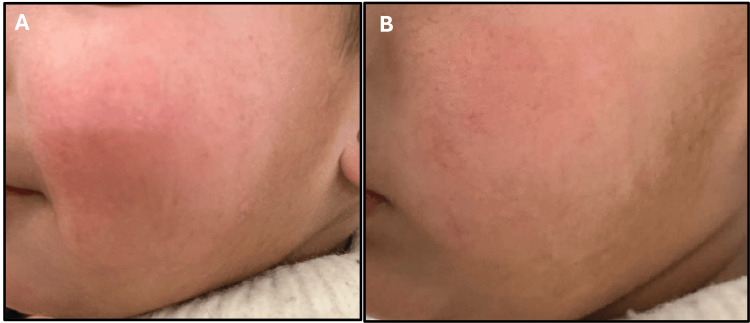
Facial atopic dermatitis before (A) and after treatment (B) showing resolution of erythema

**Figure 3:**
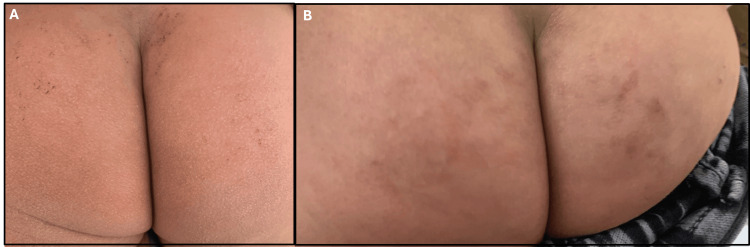
Atopic dermatitis of the gluteal region before (A) and after treatment (B) showing clinical improvement

An 18-month-old male patient presented with a six-month history of progressively worsening pruritic inflammatory dermatoses, manifesting as ill-defined erythematous xerotic patches with excoriations over the cheeks, trunk, buttocks, and flexural and extensor surfaces of the limbs. Symptoms began at 12 months of age and gradually intensified, leading to significant sleep disturbance. The course was not accompanied by fever, discharge, or systemic features, and no reproducible dietary or environmental triggers were identified. The patient had no personal or familial atopic predisposition, and antenatal, perinatal, developmental, and nutritional histories were unremarkable. Physical examination revealed an active, afebrile child with stable vitals and no extracutaneous involvement. Dermatologic assessment confirmed widespread xerotic erythematous plaques without secondary infection, with a SCORAD score of 52, consistent with severe AD. Management was initiated per the CTMP^®^ framework, incorporating a soap-free cleanser (Cetaphil Restoraderm™ Skin Restoring Bodywash), low-potency topical corticosteroid therapy (hydrocortisone 1%), intensive barrier repair with a lipid-replenishing moisturizer (Cetaphil Restoraderm™ Skin Restoring Body Moisturizer), and age-appropriate photoprotection. Treatment was planned for an eight-week duration to facilitate symptomatic control and epidermal barrier restoration, as shown in Figures 1-3). 

The CTMP^®^ framework offers a structured approach for managing AD throughout its continuum, from prevention to flare control and long-term maintenance. Integrating these pillars ensures barrier integrity, symptom control, and improved adherence [17,20,33]. 

All clinical image(s) presented in this manuscript are the property of the contributing clinician. The images have been included with the clinician’s approval, and written informed consent was obtained from the patient prior to publication.

Limitations

This review is limited by its narrative design, heterogeneity of available skincare studies, and limited India-specific quantitative implementation data. Many studies focus on individual formulations, making broad generalizations difficult. Supportive skincare remains an important adjunct to pharmacologic therapy, and its application should be individualized according to patient context and clinical guidance.

## Conclusions

The CTMP^®^ regimen - anchored in gentle cleansing, targeted anti-inflammatory therapy, barrier-repair moisturizers, and consistent photoprotection - offers a practical, evidence-based foundation for long-term disease control in AD. Yet, socioeconomic inequities, limited health and digital literacy, and restricted access to affordable skincare hinder widespread adoption in Indian settings. Strengthening clinician guidance, expanding culturally adapted patient education, and improving access to essential skincare resources are critical. Embedding a simplified CTMP^®^ framework into routine practice can enhance adherence, reduce flares, and deliver sustained outcomes across diverse populations.
